# Inverse design of soft materials via a deep learning–based evolutionary strategy

**DOI:** 10.1126/sciadv.abj6731

**Published:** 2022-01-19

**Authors:** Gabriele M. Coli, Emanuele Boattini, Laura Filion, Marjolein Dijkstra

**Affiliations:** Soft Condensed Matter, Debye Institute for Nanomaterials Science, Utrecht University, Princetonplein 1, 3584 CC Utrecht, Netherlands.

## Abstract

Colloidal self-assembly—the spontaneous organization of colloids into ordered structures—has been considered key to produce next-generation materials. However, the present-day staggering variety of colloidal building blocks and the limitless number of thermodynamic conditions make a systematic exploration intractable. The true challenge in this field is to turn this logic around and to develop a robust, versatile algorithm to inverse design colloids that self-assemble into a target structure. Here, we introduce a generic inverse design method to efficiently reverse-engineer crystals, quasicrystals, and liquid crystals by targeting their diffraction patterns. Our algorithm relies on the synergetic use of an evolutionary strategy for parameter optimization, and a convolutional neural network as an order parameter, and provides a way forward for the inverse design of experimentally feasible colloidal interactions, specifically optimized to stabilize the desired structure.

## Teaser

Machine learning autonomously designs particles that spontaneously self-assemble into desired phases of matter.

## INTRODUCTION

Self-assembly of colloidal particles is ubiquitous in nature and is considered to be of paramount importance for the design of novel functional materials. For example, viruses, lipid bilayers, tissues, atomic and molecular crystals, liquid crystals, and nanoparticle superlattices are all self-assembled from smaller components in a highly intricate way. The structure of such an assembly is determined by the interactions of the building blocks and by the thermodynamic conditions, e.g., pressure, temperature, or composition. Understanding the relation between building blocks and self-assembled arrangements is essential for materials design, as the physical properties of materials are intimately related to the structure.

On the other hand, huge progress has been made over the past decades in the synthesis and fabrication of colloidal particles, resulting in a spectacular variety of novel colloidal building blocks to the point where particles with a vast array of shapes and interaction potentials can be made on demand (*1*–*5*). Traditionally, tremendous efforts have been devoted to the “forward design” problem: Which structures with what properties are formed for a given colloidal building block under what circumstances? A major drawback of this approach is that the number of possible building blocks and thermodynamic conditions is intractably large, making a systematic exploration of these design spaces extremely demanding.

The true challenge in materials science is to develop a robust, versatile algorithm for solving the “inverse design” problem and to design building blocks that self-assemble into a target structure. The lack of such an inverse design method (IDM) forms a substantial obstacle for the full exploitation of colloidal self-assembly in the development of tomorrow’s materials (*6*–*10*).

In this work, we present a general IDM based on deep learning techniques to reverse-engineer a multitude of thermodynamic phases, ranging from crystals to liquid crystals and even quasicrystals (QCs). A machine learning–based order parameter is combined with an evolutionary strategy that searches the multidimensional parameter space to optimize the colloidal interactions and thermodynamic conditions (density, temperature, etc.) for the self-assembly of a target phase.

Designing an IDM to reverse-engineer phases, from crystals to liquid crystals and QCs, generally requires two ingredients. First, one should define an order parameter that is sensitive to the global structure of a multitude of phases and can be exploited as a fitness function indicating how “close” one is to the desired outcome. Second, one has to devise a mathematical scheme to update the design parameters based on the chosen fitness function.

The latter requirement can be easily satisfied by choosing among several techniques, either borrowed from classical optimization algorithms (*11*–*13*) or inspired by statistical physics (*12*,*14*,*15*). Our IDM uses the covariance matrix adaptation evolutionary strategy (CMA-ES) for parameter optimization (*12*, *16*).

Conversely, the choice of an effective fitness function represents the real bottleneck for any IDM to succeed. In the last decade, a plethora of order parameters has been used to define fitness functions for all kinds of phases. For instance, free-energy or chemical-potential differences with respect to the competing structures have been used to reverse-engineer three-dimensional (3D) crystal lattices starting from (non)spherical colloids (*17*,*18*). Often, full knowledge of the target crystal has been translated into a fitness function by computing the mean square displacements of the particles with respect to their target lattice points (*6*) or through the radial distribution function (*19*–*21*). The sometimes unrealistic resulting potentials have been explicitly filtered by Adorf *et al.* (*22*) to obtain smooth and short-range interactions.

Although all these fitness function definitions brilliantly achieve their goals, they often lack generality, and they are not able to simultaneously and equally penalize competing phases. In other words, they do not have the ability to create an approximately flat fitness landscape, where the design engine can move smoothly, with only one preferred region corresponding to the target phase. Moreover, in the case of QCs, despite the certified need of two inherent length scales in the system (*23*–*26*), the actual positions of the constituent particles remain unknown, therefore representing a substantial challenge to the above strategies.

**Fig. 1.**
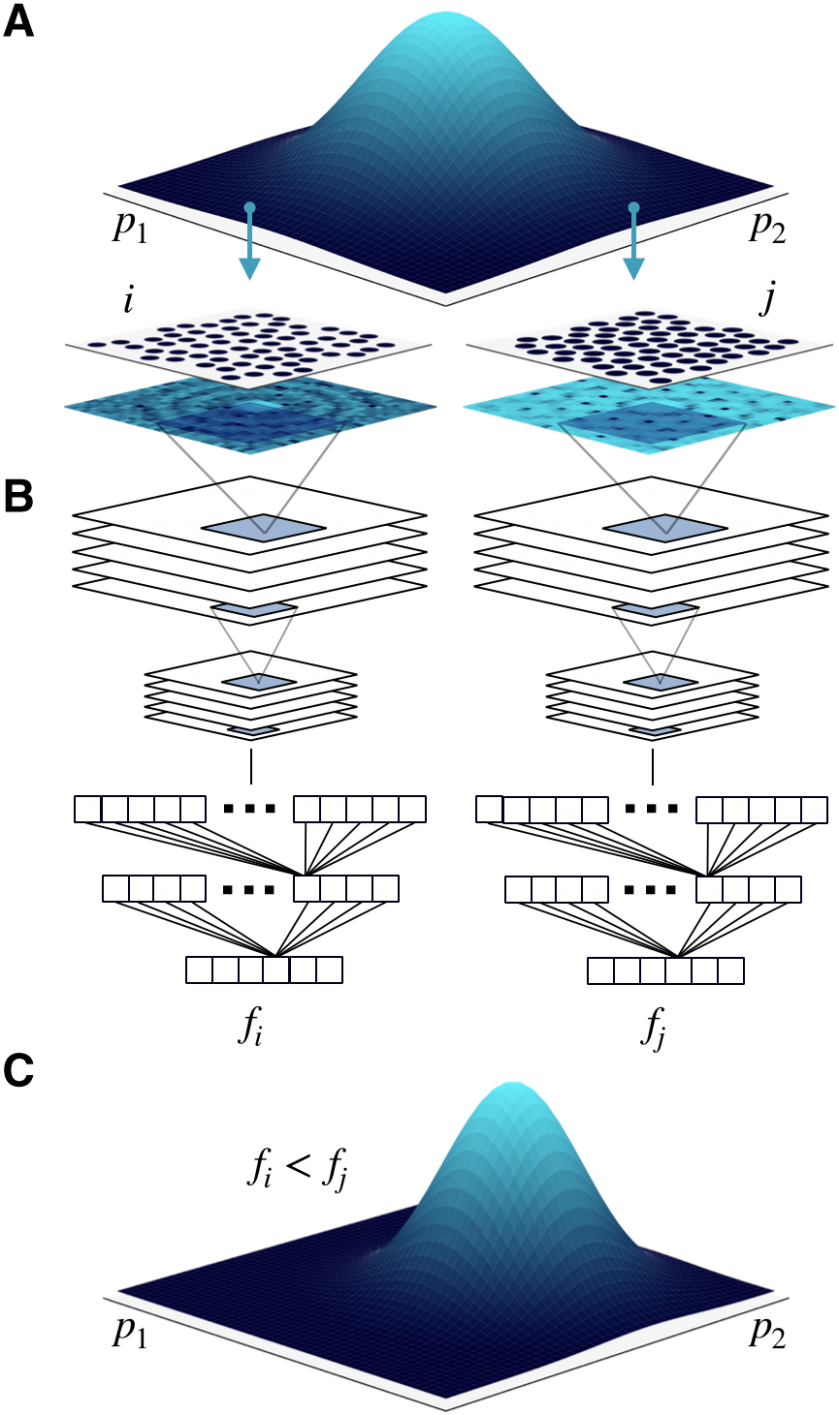
Schematic representation of the three steps performed at each generation. (**A**) In the first step, we draw candidate sets of parameters (*p*_1_ and *p*_2_ in the figure) from a multivariate Gaussian distribution. For each set or sample, we then perform a simulation. (**B**) In the second step, samples are ranked and scored on the basis of their fitness *f*, which is evaluated using a convolutional neural network trained to classify phases according to their diffraction patterns. Samples with a higher likelihood of being classified as the target phase will be scored with a higher fitness. (**C**) In the third and final step, the Gaussian distribution is updated to move toward regions of the parameter space where the fittest samples have been encountered.

Inspired by the highly successful history of identifying phases by their scattering patterns in combination with advances in machine learning, we attack the problem from a new avenue and directly use an encoding of the structure factor as the order parameter. To this end, we train a convolutional neural network (CNN) to classify different phases from their diffraction pattern, and use the result to construct a fitness function, such that configurations with a higher likelihood of being classified as the target phase will be scored with a higher fitness. A sketch of the final algorithm is shown in Fig. 1. A detailed discussion on the choice of a CNN-based fitness function can be found in the Supplementary Materials. This algorithm turns out to be extremely robust and versatile, facilitating the inverse design of not only crystal and liquid crystalline phases but also QCs, which due to their nonperiodicity are notoriously difficult to inverse design.

## RESULTS

### Our IDM

Our IDM combines the CMA-ES for parameters optimization and a CNN for the fitness evaluation, which are both described in detail in Methods. The goal is to optimize the free parameters of a given model to favor the formation of a target phase.

The method proceeds in generations, or iterations, consisting of essentially three steps: (i) sampling, (ii) fitness evaluation, and (iii) update. In the following, we give a general overview of these three steps, which are sketched in Fig. 1.

In the first step (Fig. 1A), we draw a fixed number of candidate sets of parameters from a multivariate Gaussian distribution. The dimension of this multivariate Gaussian distribution is determined by the number of design parameters that we wish to tune. For each candidate set of parameters, we then perform a simulation of the system and save a number of representative configurations. In the second step (Fig. 1B), we score and rank the samples based on their fitness *f*. In general, the fitness is a measure of similarity between a sample and a specific target, and it is maximized when the target is reached. Here, we introduce a new fitness function based on CNNs that are trained to classify different phases based on their diffraction patterns. We use this CNN to process the configurations saved during each simulation and assign a larger fitness to samples with a higher probability of being classified as the target phase. Last, on the basis of this score, the mean and the covariance matrix of the multivariate Gaussian distribution are updated using the CMA equations, which are designed to facilitate an efficient exploration of parameter space. As sketched in Fig. 1C, the update not only allows the mean of the distribution to move toward regions with a higher fitness but also speeds up sampling by stretching the distribution when several updates are in the same direction and then shrinking it once the fitness is maximized. This whole procedure is repeated multiple times until the fitness is maximized and/or a predetermined convergence criterion is met.

### Setting up the IDM in two dimensions

The first model we consider is a 2D system in which the particles interact with a hard-core square-shoulder (HCSS) potential

$$\beta u(r)=\{\begin{array}{cc} \infty , & r<\sigma \\ \beta \epsilon , & \sigma \leq r\leq \delta \\ 0, & r>\delta \end{array}$$ (1)

where *r* is the center-of-mass distance between two particles, ϵ is the interaction strength, σ is the core diameter, δ is the interaction range, and β = 1/*k*_B_*T*, with *k*_B_ as Boltzmann’s constant and *T* as the temperature. This model has been shown to self-assemble into a variety of phases (*24*, *27*–*29*), including several crystal structures and various QCs, which makes it an ideal playground for setting up and testing our IDM. The three QCs we consider here, which are the dodecagonal QC (QC12), the decagonal QC (QC10), and the octadecagonal QC (QC18), are found to be stable for different values of the interaction range δ and only in a tiny range of densities ρ and temperatures *T*. In all cases we explore, the competing stable phases include the fluid, the hexagonal (HEX) crystal, and the square (SQ) crystal phase.

To set up our IDM, we trained a CNN to classify the aforementioned phases based on their 2D diffraction patterns, as described in Methods. Specifically, the CNN takes as input the diffraction pattern of a given configuration and outputs a vector of real numbers with as many components as the number of phases to distinguish. Each number in the output is indicative of the likelihood that the given input corresponds to one of the phases. This output is then used to define the fitness function to target a specific phase.

The dataset for training the CNN is built by performing Monte Carlo simulations of the HCSS model in the *NPT* ensemble. For each phase, we perform simulations at different state points and collect a large number of independent configurations. The set of diffraction patterns generated from these configurations constitutes the dataset on which the CNN is trained and validated. Overall, we find the CNN to be highly effective and able to classify all phases with 100% accuracy.

### Reverse engineering of QC12 in the HCSS model

**Fig. 2.**
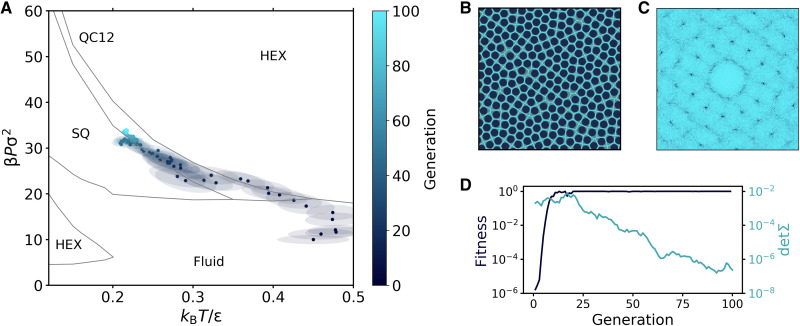
Reverse engineering of QC12 in the HCSS model. (**A**) Evolution of the Gaussian distribution in the *k*_B_*T*/ϵ − β*P*σ^2^ plane. Points and ellipses represent the mean and the covariance matrix (within one SD) of the distribution. The phase diagram in the background is constructed using data points from (*27*). (**B**) Representative snapshot of QC12 obtained during the last generation. The hard cores are shown in a dark color, while lines show their Voronoi tessellation. (**C**) Diffraction pattern of the snapshot in (B). (**D**) Evolution of the mean fitness and the determinant of the covariance matrix.

We start our investigation by considering the HCSS model with a fixed value of the shoulder width δ = 1.4σ, at which the QC12 phase has been shown to be stable (*27*, *28*). The phase diagram as a function of temperature and pressure [constructed using data points from (*27*)] is reported in Fig. 2A.

The goal here is to reverse-engineer the QC12 phase by letting the evolutionary strategy find the narrow region in the phase diagram where the QC12 phase is stable by tuning the system parameters pressure *P* and temperature *T*. In other words, we keep the interaction parameters fixed while trying to optimize the thermodynamics variables to favor the formation of QC12. Our knowledge of the phase diagram allows us to easily assess and monitor the performance of the reverse engineering process.

To explicitly target QC12, we use the output of the trained CNN to define the fitness function *f* for the evolutionary strategy. In particular, for any sample, i.e., for any simulation, we define the fitness as $f={\bar{P}}_{\text{QC}12}$, where *P*_QC12_ is the probability that the diffraction pattern of a given configuration is classified as a QC12 by the CNN, and the bar indicates an average taken over representative configurations visited during the simulation.

The results of the reverse engineering process are summarized in Fig. 2. Starting the reverse engineering process with a Gaussian centered in the region of stability of the fluid phase, the algorithm reaches the region where the target QC12 is stable in approximately 25 generations. Figure 2A shows the evolution of the multivariate Gaussian distribution in the temperature *k*_B_*T*/ϵ-pressure β*P*σ^2^ plane across successive generations. A representative snapshot obtained in the last (100th) generation is shown in Fig. 2B, while the corresponding diffraction pattern, characterized by 12-fold rotational symmetry, is shown in Fig. 2C.

The success of the algorithm heavily relies on the ability of the CNN to spot even small structural variations in the system. At the early stages of the reverse engineering process, when the system is in the fluid phase, the algorithm already finds it convenient to increase the pressure and, hence, the density to increase the overall structural order. This can clearly be seen in Fig. 2D, where we plot the evolution of the mean fitness averaged over all samples. Although the variations of the fitness in the early generations are very tiny, they are sufficient to guide the evolutionary strategy in the right direction.

An efficient exploration of phase space is then made possible by the CMA equations, which evolve the Gaussian distribution at each generation. This not only allows the mean of the distribution to move toward regions with a higher fitness but also allows the covariance to stretch when several updates are in the same direction and then shrink once the fitness is maximized. This is shown in Fig. 2D, where we plot the evolution of both the mean fitness and the determinant of the covariance matrix. The determinant becomes larger when the fitness improves, and it decays exponentially once the fitness is maximized.

Note that, here, we initialized the mean of the Gaussian distribution at a specific state point within the region of stability of the fluid phase, but we find the algorithm to be largely robust to changes in the initial conditions. In the Supplementary Materials, we show additional trajectories of the reverse engineering of QC12 obtained by starting with a Gaussian distribution centered at different state points, i.e., in the fluid phase, the SQ phase, the HEX phase at relatively high temperature and low pressure, and the HEX phase at relatively low temperature and high pressure. In all cases, the mean of the parameter distribution converges to the region of stability of the target QC12, showing that the performance is not affected by the particular choice made for the initial conditions.

Furthermore, we would like to stress a crucial aspect that demonstrates the versatility of the algorithm. The same method, and the exact same CNN, can be used to target any phase that was included in the training dataset, simply by changing the definition of the fitness. For instance, to reverse-engineer the HEX crystal phase, it is sufficient to impose $f={\bar{P}}_{\text{HEX}}$. A trajectory of the reverse engineering of the HEX crystal is shown in the Supplementary Materials.

### Reverse engineering of QC12, QC10, and QC18 in the HCSS model

**Fig. 3.**
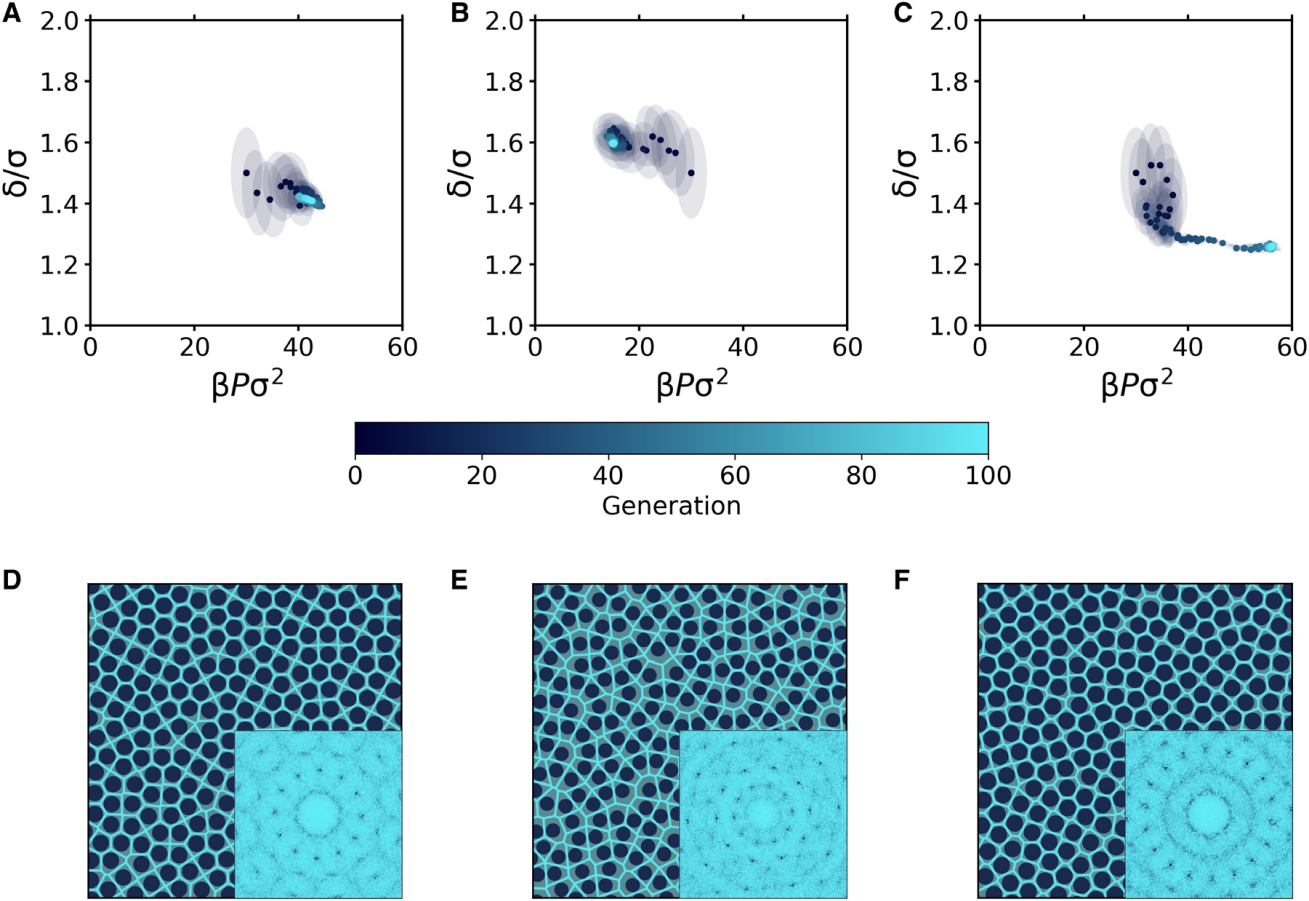
Reverse engineering of QC12, QC10, and QC18 in the HCSS model. (**A** to **C**) Evolution of the Gaussian distribution in the β*P*σ^2^ − δ/σ plane during the reverse engineering of the (A) QC12, (B) QC10, and (C) QC18 phases. Points and ellipses represent the mean and the covariance matrix (within one SD) of the distribution. (**D** to **F**) Representative snapshots of (D) QC12, (E) QC10, and (F) QC18 obtained in the last generation, along with their diffraction patterns and Voronoi tessellations.

As already discussed, in addition to QC12, the HCSS model exhibits two other quasicrystalline structures, which are stabilized for different values of the shoulder width δ. As a natural next test, we now explore whether we can reverse-engineer all the three stable QCs (QC12, QC10, and QC12) considered in this work. To this end, we fix the temperature to *k*_B_*T*/ϵ = 0.17, a temperature for which all three QCs are stable, and let the evolutionary strategy optimize the shoulder width δ and the pressure *P* for each specific QC. In all three cases, we start the reverse engineering process from the same state point in the fluid phase (δ = 1.5σ and β*P*σ^2^ = 30) and choose the fitness function appropriate for the target phase. The results of the reverse engineering process are summarized in Fig. 3. In particular, Fig. 3 (A to C) shows the evolution of the multivariate Gaussian distribution when targeting (i) QC12, (ii) QC10, and (iii) QC18. Depending on the QC to be found, the distribution evolves in different directions and eventually converges to different state points. In all cases, the final values of pressure and shoulder width obtained are in excellent agreement with those at which the three QCs have been shown to be stable (*24*, *27*, *28*, *29*). Representative snapshots of the QCs were obtained, and their diffraction patterns are shown in Fig. 3 (D to F). Each diffraction pattern immediately confirms the presence of the correct quasicrystalline structure.

### Application to a new model interaction

Thus far, we have only addressed the model that was used for training the CNN. A natural next question is whether the method is general enough to work on other model systems without having to retrain the CNN for the specific model under consideration. To answer this question, we now consider a 2D softened-core-shoulder (SCS) model with an interaction potential given by

$$u(r)/\epsilon ={\left(\frac{\sigma }{r}\right)}^{14}+\frac{1-\text{tanh}\;[k(r-\delta )]}{2}$$ (2)

where ϵ is the energy scale, σ represents the typical core diameter, and *k* and δ are two parameters that, respectively, control the steepness and the characteristic interaction range. Similar to the HCSS, QC12 has been shown to be stable in a limited range of densities and temperatures with a shoulder width of δ = 1.35σ and *k*σ = 10 (*30*, *31*).

**Fig. 4.**
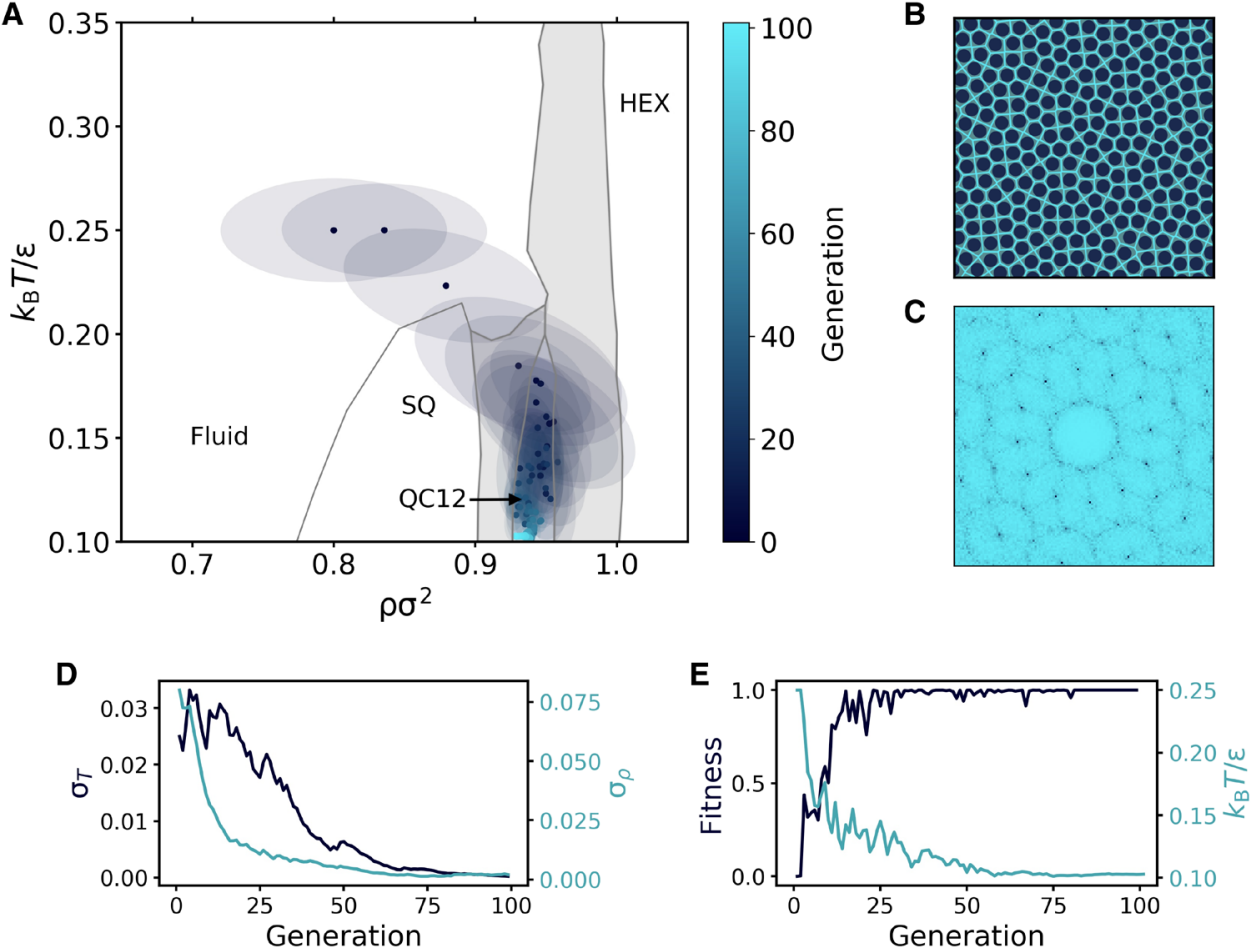
Reverse engineering of QC12 in the SCS model. (**A**) Evolution of the Gaussian distribution in the ρσ^2^ − *k_B_T*/ϵ plane. Points and ellipses represent the mean and the covariance matrix (within one SD) of the distribution. The phase diagram in the background is constructed using data points from (*30*). Coexistence regions are indicated in light gray. (**B**) Representative snapshot of QC12 obtained during the last generation and its Voronoi tessellation. (**C**) Diffraction pattern of the snapshot in (B). (**D**) Evolution of the square root of the covariance matrix’s diagonal elements, which correspond to the SDs along the temperature (σ*_T_*) and density (σ*_ρ_*) directions. (**E**) Evolution of the mean fitness and the mean temperature in (A).

To test the ability of our method to be effective on new types of interactions, we use the same CNN that was trained on the HCSS model to reverse-engineer QC12 in the SCS model. Similar to the HCSS case, we keep the interaction parameters fixed, i.e., δ = 1.35σ and *k*σ = 10, and let the evolutionary strategy find the region of densities and temperatures in which QC12 is stable. The phase diagram in Fig. 4A is used as a reference to assess and monitor the performance of the method. Note that, since this phase diagram is in terms of density and temperature, simulations are now performed in the canonical ensemble. Moreover, in contrast to the HCSS case, there are now stable coexistence regions between multiple phases (indicated with a gray background in Fig. 4A). As the CNN was not trained on configurations with a phase coexistence, this represents a further robustness test for our method.

The results of the reverse engineering process are summarized in Fig. 4. Specifically, Fig. 4A shows the evolution of the multivariate Gaussian distribution in the temperature-density plane. Starting with a distribution centered in the fluid region, the algorithm immediately starts to increase the density and lower the temperature to increase the overall order. Impressively, after only five generations, the mean of the distribution is already inside the region of stability of QC12, demonstrating the robustness of the CNN to changes in the interaction potential. In the remaining generations, the covariance of the distribution shrinks, and the mean moves toward lower temperatures in the phase diagram. A representative snapshot of QC12 obtained during the last generation and its diffraction pattern are shown in Fig. 4 (B and C), respectively.

Looking more closely at the evolution of the model parameters, it is interesting to observe the different behavior of the temperature and density components. After the first five iterations, the density simply oscillates in the tiny range of stability of QC12, while a large exploration keeps happening in temperature. This can be seen also by looking at the evolution of the SDs of temperature (σ*_T_*) and density (σ*_ρ_*) in Fig. 4D. While σ_ρ_ decays almost monotonously from the very beginning, σ*_T_* oscillates for about 20 generations before starting its decay.

We would also like to stress that the reason why the algorithm seems to prefer lower temperatures, despite being already in the stability region of the target phase, is solely related to the nature of the CMA-ES equations (see Methods) and is not a feature of the selected fitness function. A detailed discussion of this behavior can be found in the Supplementary Materials

.

### Phase discovery

The fundamental ability of the algorithm to generalize to different interaction potentials opens up the possibility of finding quasicrystals in new model systems. For instance, given the similarities between the SCS and the HCSS models, we might ask whether also the SCS model stabilizes different QCs for different values of the shoulder width δ. We note that, compared to the HCSS model, much less is known about the phase behavior of the 2D SCS system.

Here, we explore the possibility of the SCS model to form a QC10. To this end, we fix *k*σ = 10 as in the previous case, and let the evolutionary strategy optimize three parameters: shoulder width δ, temperature *T*, and pressure *P*. Note that, by varying these three parameters simultaneously, the algorithm might encounter phases that were not included in the dataset for training the CNN. We do not expect this to be a problem, as long as no phase is misclassified as the target phase. This could possibly cause the algorithm to get stuck and eventually converge to the wrong phase. While this problem did not occur in our test, a simple solution would be to include the newly found phase in the training dataset and retrain the CNN.

**Fig. 5.**
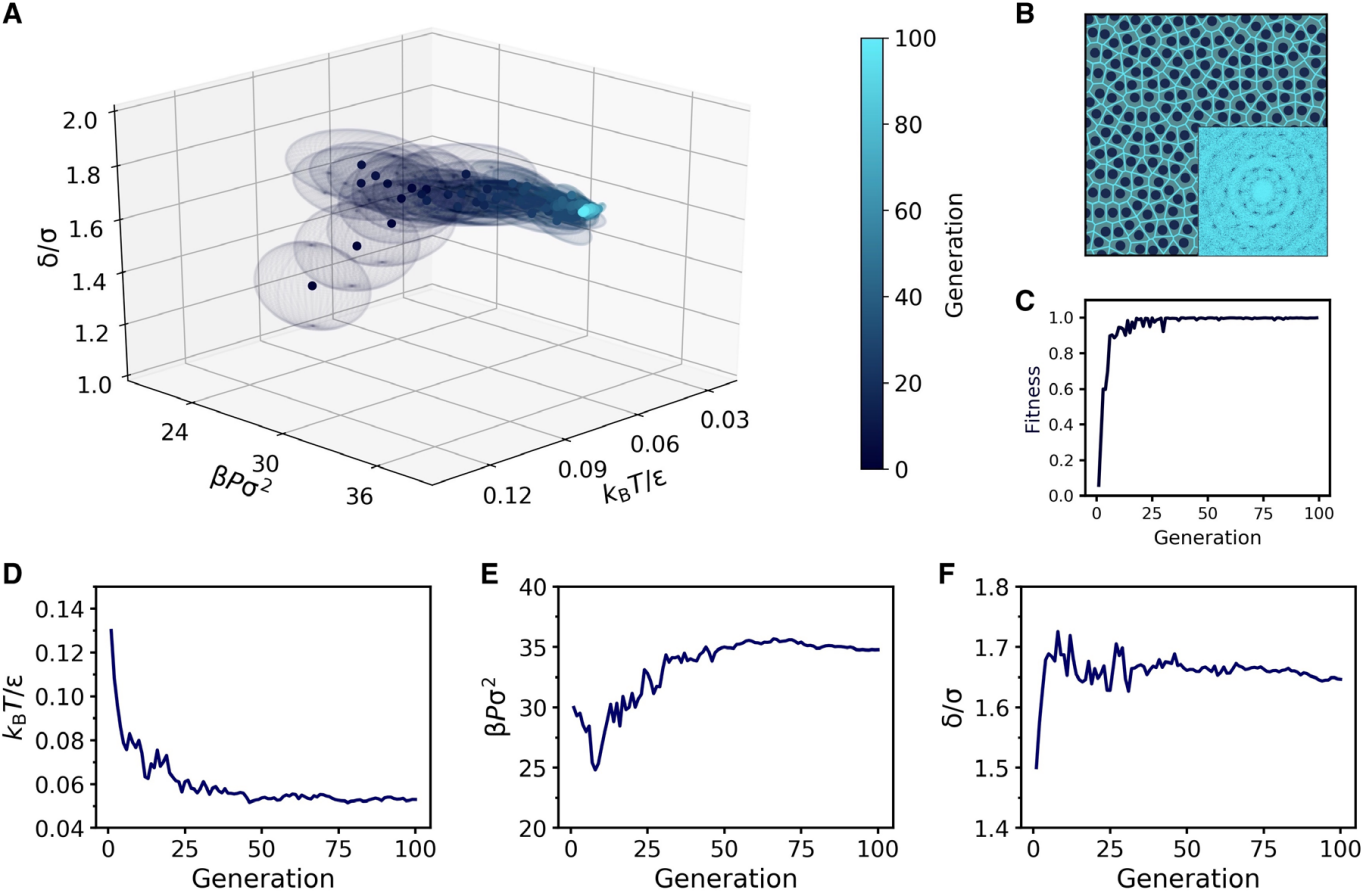
Discovery of QC10 in the SCS model. (**A**) Evolution of the Gaussian distribution in *k*_B_*T*/ϵ − β*P*σ^2^ − δ/σ space. Points and ellipsoids represent the mean and the covariance matrix (within one SD) of the distribution. (**B**) Representative snapshot of QC10 obtained during the last generation, along with its diffraction pattern and Voronoi tessellation. (**C**) Evolution of the mean fitness. (**D** to **F**) Evolution of the three parameters in (A) optimized in the reverse engineering process: (D) temperature *k*_B_*T*/ϵ, (E) pressure β*P*σ^2^, and (F) shoulder width δ/σ.

The results of the reverse engineering process are summarized in Fig. 5. Starting from a fluid phase, the evolutionary strategy decreases the temperature and increases both the pressure and shoulder width to maximize the fitness (see Fig. 5, C to F), finding the not-yet-predicted QC10 phase for this system. As a further confirmation that the algorithm has found a QC10, Fig. 5B shows a representative snapshot obtained during the last generation, along with the corresponding diffraction pattern. Hence, our algorithm has successfully located a new phase in the SCS model.

### Extension to 3D systems

Up to this point, we have shown the efficacy of our method for 2D systems where the scattering pattern is simply a 2D image. Last, we extend and test our approach on 3D systems. To do so, we consider a 3D system of rod-like particles, modeled as hard-core spherocylinders with a soft deformable corona. We consider spherocylinders with a length-to-diameter ratio *L*/σ = 5, interacting via the pair potential in Eq. 2, where the center-of-mass distance *r* is replaced by the minimum distance between two rods *d_m_*. Note that *d_m_* depends on both the center-of-mass distance and the relative orientation of the two rods.

**Fig. 6.**
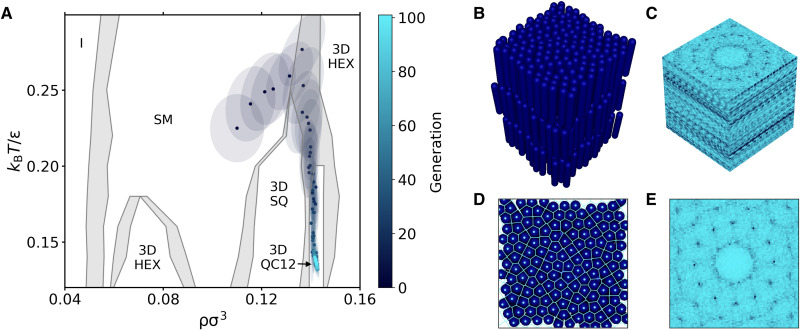
Reverse engineering of QC12 in a 3D model of soft spherocylinders. (**A**) Evolution of the Gaussian distribution in the ρσ^3^ − *k_B_T*/ϵ plane. Points and ellipses represent the mean and the covariance matrix (within one SD) of the distribution. The phase diagram in the background is constructed using data points from (*32*). Coexistence regions are indicated in light gray. (**B**) Representative snapshot of the 3DQC12 obtained during the last generation and (**C**) its 3D diffraction pattern. (**D**) Top view of the snapshot in (B). The centers of mass and the corresponding Voronoi tessellation are highlighted in a light color. (**E**) In-layer diffraction pattern of the top view in (D).

The phase behavior of this system with *k*σ = 10 and δ = 1.35σ has been recently studied in (*32*). In addition to the standard isotropic (I) and smectic (SM) phases, this model has been shown to stabilize phases consisting of quasi-2D layers with unconventional symmetries, including SQ (3DSQ) and HEX (3DHEX) crystals, and a 3D 12-fold QC (3DQC12). The phase diagram in terms of density and temperature is reported in Fig. 6A.

As done in the 2D case, to set up our IDM, we train a CNN to classify all the stable phases of this system. Note, however, that the inputs of the CNN are now 3D diffraction patterns (see Methods for more details). Again, we find the CNN to be highly effective and able to classify all phases with 100% accuracy. The output of the trained CNN is then used to define the fitness for the evolutionary strategy where we target the 3DQC12 phase.

The results of the reverse engineering process are summarized in Fig. 6. In particular, Fig. 6A shows the evolution of the multivariate Gaussian distribution in the density-temperature plane. Starting with a distribution centered in the SM phase, the mean of the distribution evolves via the coexistence region of the SM and 3DHEX phase, to the 3DSQ-3DQC12 phase coexistence region, until it converges in the stability region of the 3DQC12 phase. We note that, although the shortest path in parameter space requires the distribution to cross the 3DSQ region, the algorithm actually avoids it, preferring to enter the coexistence region at high temperature and then move downward in temperature, where samples with higher fitness are encountered. Unexpectedly, this pathway for the formation of QC12 phases was also identified in (*29*).

A representative snapshot of the 3DQC12 obtained during the last generation along with its 3D diffraction pattern is shown in Fig. 6 (A and B, respectively). As a further confirmation of the in-layer QC12 arrangement, Fig. 6 (C and D) shows a top view of the same snapshot and the corresponding in-layer 2D diffraction pattern.

The extension of our method to the 3D case is of particular interest from a practical point of view. While a 2D diffraction pattern immediately provides structural information that is easy to read even by eye, the 3D counterpart is much harder to interpret. For this reason, to deal with 3D systems, it is often necessary to project the particle coordinates onto the planes with the relevant symmetries. This aspect becomes irrelevant when using a CNN that naturally processes the full 3D information because of its inherent architecture.

## DISCUSSION

Diffraction patterns are used across a multitude of areas in materials science to understand what structure one is dealing with. In general, this information constitutes a unique signature of each structure, whether it is a crystal, a fluid, a liquid crystal, or a QC, and shows notable robustness to changes in density and interaction potentials. This can efficiently incorporate all the relevant information of a target phase and therefore provides a natural order parameter for IDMs.

With the present work, we have shown how the use of CNNs as diffraction patterns classifiers can provide a useful order parameter for the reverse engineering of a multitude of phases. For the above reason, an IDM built on such an order parameter is not restricted to a specific class of materials but is instead naturally tailored to reverse-engineer multiple colloidal phases, ranging from crystals and QCs to liquid crystals.

Our results pave the way to structure optimization and discovery, especially with binary and ternary systems, where the design space becomes even larger due to new system parameters such as size ratio and composition. In these cases, where the present knowledge of phase diagrams and emerging phases is limited, IDMs can prove extremely precious and efficient.

## METHODS

### CNNs as a fitness function

CNNs are a particular type of deep neural networks specifically designed to handle tensorial inputs, such as images. For a detailed description of CNNs, see, e.g., (*33*). In this work, we train a CNN to classify different phases from their diffraction patterns, which are either 2D or 3D images. The output of the CNN is then used to define a fitness function *f* for the evolutionary strategy.

More specifically, the CNN takes as input the diffraction pattern of a given configuration and outputs a vector of real numbers with as many components as the number of phases to distinguish. Each number in the output is indicative of the probability that the given input corresponds to one of the phases. We use this CNN to process the configurations saved during each simulation and define the fitness of a given sample as

$$f={\bar{P}}_{\text{target}}$$ (3)

where *P*_target_ is the probability that the diffraction pattern of a given configuration is classified as the target phase by the CNN, and the bar indicates an average taken over 10 representative configurations saved during the simulation of that sample.

### Training the CNNs

To train the CNNs to recognize different phases, we need to perform a number of different steps. Specifically, we first generate a number of real-space equilibrium configurations for each phase and then generate the associated diffraction patterns. To reduce computational time and memory usage, these diffraction patterns are preprocessed before being used to train the CNNs. Each of these steps is described in detail in the remainder of this section.

### Generating the training configurations

The configurations for training the CNNs are generated by performing Monte Carlo simulations of the 2D HCSS model (*24*, *27*, *28*, *29*) and the SCS model of spherocylinders in three dimensions (3D) (*32*). In 2D, simulations are performed in the isobaric-isothermal ensemble (*NPT*) of a system of *N* = 256 particles in a square box of side length *L* with periodic boundary conditions. One volume move is performed every *N* particle displacement moves. The maximum displacement and the maximum volume change are tuned during the equilibration steps to obtain acceptance ratios of 45 and 20%, respectively.

For each of the six phases considered (fluid, HEX, SQ, QC12, QC10, and QC18), we run simulations at different state points. All simulations are equilibrated for a total of 5 × 10^5^ Monte Carlo sweeps. The equilibration phase is followed by a total of 1 × 10^6^ sweeps, during which we save a configuration every 10^3^ sweeps (yielding 10^3^ independent configurations). This is repeated for 10 different state points for each of the considered phases.

In 3D, simulations are performed in the canonical ensemble (*NVT*) of a system of *N* = 432 particles in a rectangular box elongated in the *z* direction (i.e., *L_x_* = *L_y_* = *L* and *L_z_* > *L*) and with periodic boundary conditions. The maximum displacement is again tuned during the equilibration steps to obtain an acceptance ratio of 45%.

For each of the five phases considered (I, SM, 3DHEX, 3DSQ, and 3DQC12), we run simulations at different state points, with the same number of production sweeps. However, the duration of the equilibration phase is 5 × 10^4^ Monte Carlo sweeps, and five state points are taken into account for each of the considered phases.

### Generating the diffraction patterns

Diffraction patterns for each configuration are evaluated using

$$S(k)=\frac{1}{N}\rho (k)\rho (-k)$$ (4)

where $\rho (k)=\sum_{j=1}^{N}e^{-ik\cdot r_{j}}$ is the Fourier transform of the density, *r_j_* is the position of particle *j*, and **k** is a wave vector. In 2D, the **k** vectors are chosen by $k=\frac{2\pi }{L}(n_{x},n_{y})$, where *n_x_* and *n_y_* are two integers in the interval [ − 64,64]. As a result, the 2D diffraction patterns considered in this work are built on a 129 × 129 grid. In 3D, the **k** vectors are chosen by $k=2\pi (\frac{n_{x}}{L},\frac{n_{y}}{L},\frac{n_{z}}{L_{z}})$, with *n_x_*, *n_y_*, *n_x_* ∈ [−32,32], resulting in a 65 × 65 × 65 grid.

**Fig. 7.**
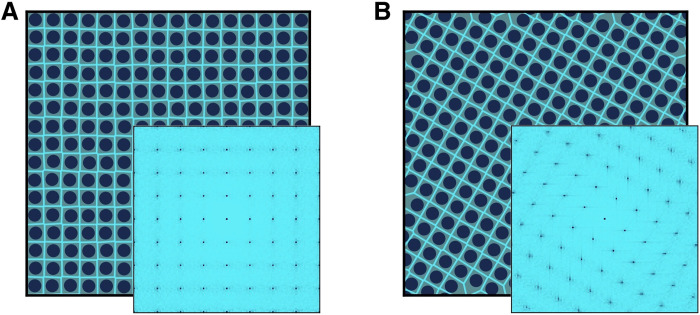
Data transformation. (**A**) Snapshot and diffraction pattern of an SQ crystal in its original orientation. (**B**) Same snapshot and diffraction pattern as (A) after a rotation by a π/6 angle. Note that the rotation is performed in real space.

While diffraction patterns are, by definition, translationally invariant, they are not invariant to rotations. However, we must ensure that the CNNs are able to classify the desired phases regardless of their orientation. To this end, each training configuration is rotated by a random angle before evaluating its diffraction pattern. A representation of this transformation in the 2D case is shown in Fig. 7. In the 3D case, given the inherent symmetry of the model of spherocylinders considered, we randomly rotate each configuration around the *z* axis (which always corresponds to the elongated axis of the box). In a more general case, one could perform random rotations around a randomly selected axis. Note that, to rotate a configuration, we first create a larger copy of the system by copying the original simulation box in all directions. We then rotate this larger copy of the system and finally take a portion of it with the same volume as the original simulation box. Note that the retained portion might have a slightly different number of particles than the original configuration. The sets of diffraction patterns obtained after having rotated each configuration are finally used to build the datasets for training the CNNs.

### Preprocessing

**Fig. 8.**
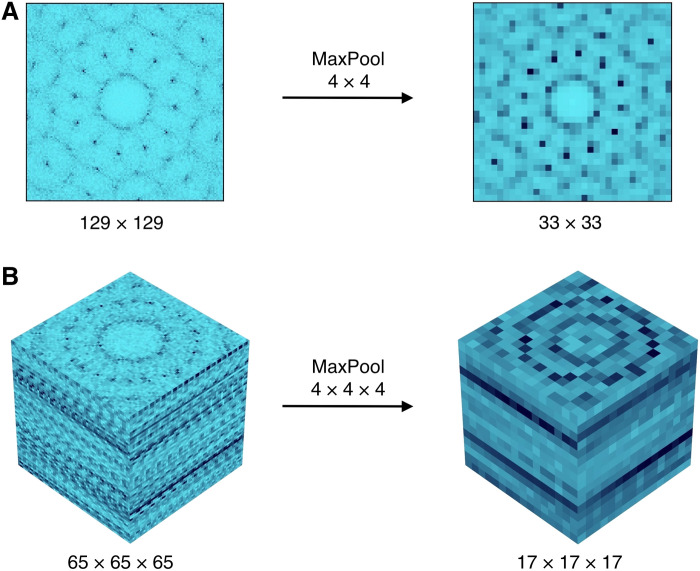
Preprocessing. The size of the diffraction pattern of a QC12 in (**A**) 2D and (**B**) 3D is reduced through a MaxPooling filter.

To increase the overall efficiency, the diffraction patterns undergo a final preprocessing step before being used as the input of the CNNs. In particular, each diffraction pattern passes through a MaxPooling filter (with size 4 × 4, zero-padding with size *p* = 2, and stride *s* = 4) that effectively reduces the input size by a factor of 4 in each dimension. The effect of this transformation is shown in Fig. 8 for both the (i) 2D and (ii) 3D cases.

Note that this is not a necessary step of the algorithm, and its only purpose is to increase the efficiency of the method in terms of computational time and memory usage. With such a preprocessing, the CNNs used here can be trained within 1 hour on the central processing unit (CPU) of a modern laptop.

### Neural network architecture

**Fig. 9.**
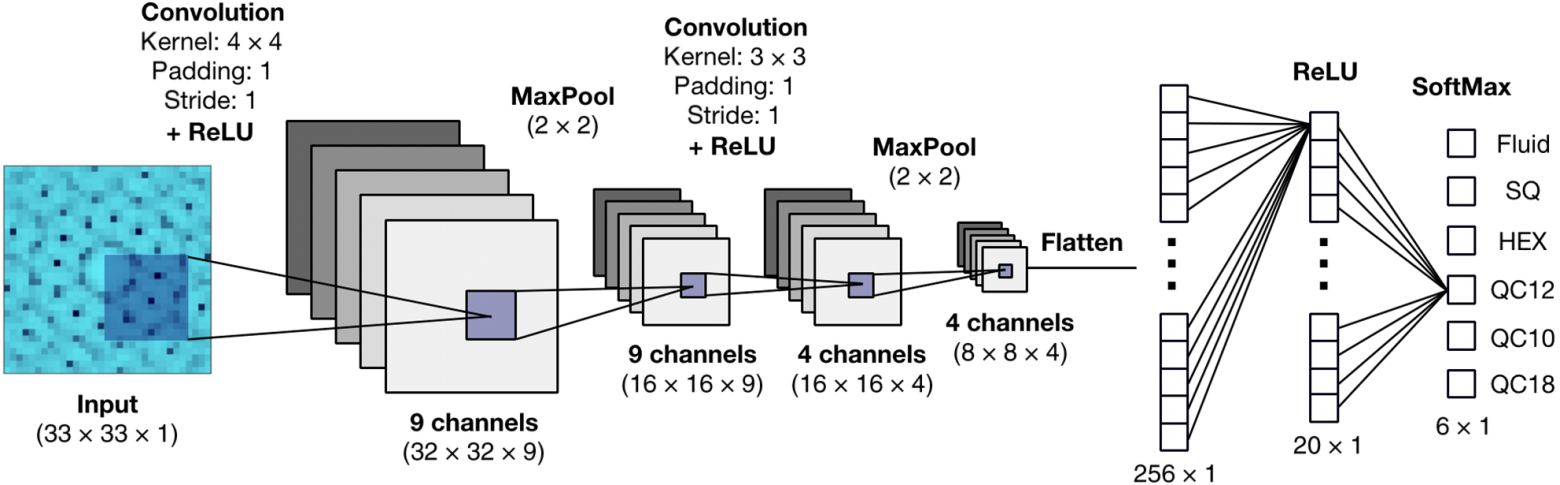
Representation of the 2D convolutional neural network. The network is composed of two convolutional layers for feature extraction and a fully connected part with one hidden layer for the final classification. All details about kernels, layer size, and activation functions are also shown.

The CNNs used in this work are composed of two convolutional layers for feature extraction and a fully connected part with one hidden layer for the final classification. The architecture of the 2D CNN is shown in Fig. 9. As shown in the figure, each convolutional layer performs three operations on the input: a convolution, a nonlinear transformation through a ReLU activation function (where ReLU(x) = max(0,x)), and a downsampling operation through a 2 × 2 MaxPooling layer (with padding size *p* = 0 and stride *s* = 2). In the following, we give all the details about the network parameters.

The first convolutional layer has one input channel (i.e., the diffraction pattern to process) and nine output channels (i.e., the extracted features). As indicated in Fig. 9, the kernels used in this layer have size 4 × 4, padding *p* = 1, and stride *s* = 1. The second convolutional layer has nine input channels and four output channels, and the kernels of this layer have size 3 × 3, padding *p* = 1, and stride *s* = 1. The output of the second convolutional layer is stacked and flattened to be used as the input of the fully connected part of the network. The latter consists of a hidden layer of dimension 20 with a ReLU activation function and an output layer with a SoftMax activation function. The size of the output layer is equal to the number of phases we wish to distinguish, which is six in the 2D case.

The 3D CNN has almost the same structure as the 2D one, with the only exception being that the convolutional kernels are extended to three dimensions (e.g., a 3 × 3 kernel in 2D becomes a 3 × 3 × 3 kernel in 3D), and the output layer has a dimension of 5 (we consider five phases in the 3D system).

### Training

The parameters of the CNNs are optimized by minimizing the cross-entropy loss with the addition of a weight decay regularization term (*34*, *35*). Specifically, the loss is minimized with the Adam optimizer (*36*), a learning rate of 10^−4^, and a PyTorch implementation (*37*). Early stopping is also applied to prevent overfitting.

### Workflow of the CMA-ES

The CMA-ES optimizes iteratively the design parameters across successive generations. At each generation, we draw *n* samples from a multivariate Gaussian distribution, whose dimension *D* corresponds to the number of parameters we wish to optimize. Subsequently, we evaluate the fitness function *f* of the generated samples, we order the samples in ascending order based on their fitness, and we pick the set **X** of the best *k* samples. Last, the mean $\vec{\mu }$ (a *D*-dimensional vector) and the covariance matrix Σ = σ^2^**C** of the Gaussian distribution are updated using the following equations

$$\begin{array}{c} \mu_{i}\prime =\mu_{i}+\sum_{x\in X}w(x)(\lambda_{i}(x)-\mu_{i})\\ q_{i}\prime =(1-c_{1})q_{i}+c_{2}{\sqrt{\Sigma^{-1}}}_{\mathrm{ij}}(\mu_{j}\prime -\mu_{j})\\ p_{i}\prime =(1-c_{3})p_{i}+c_{4}(\mu_{i}\prime -\mu_{i})\\ C_{\mathrm{ij}}\prime =(1-c_{5}-c_{6})C_{\mathrm{ij}}+c_{6}p_{i}\prime p_{j}\prime \\ +c_{5}\sum_{x\in X}w(x)(\frac{\lambda_{i}(x)-\mu_{i}}{\sigma }\frac{\lambda_{j}(x)-\mu_{j}}{\sigma }-C_{\mathrm{ij}})\\ \sigma \prime =\sigma \text{exp}[c_{7}(\frac{∥\vec{q\prime }∥}{<\langle ∥N(0,I)∥\rangle >}-1)] \end{array}$$ (5)

where **X** denotes the set of the *k* best samples consisting of multiple configurations obtained for *k* different parameter sets [denoted by λ*_i_*(*x*)], *w*(*x*) is the normalized distribution of weights based on the fitness of the samples, and *c_i_*’s are free parameters. We choose *w*(*x*) ∝ log (*k* + 1) − log (*m*), where *m* is the rank index of sample *x* (*m* = 1 for the configuration with the largest *f* value). $\vec{q}$ and $\vec{p}$ are additional *D*-dimensional vectors that control, respectively, the changes in amplitude and directionality of the covariance matrix. In addition, 〈∥*N*(0,*I*)∥〉 is the average length of a vector drawn from a multivariate Gaussian distribution centered in the origin and where the covariance matrix is the identity matrix. In the present work, we use *n* = 10 and *k* = 5 for all cases where we optimize two parameters, i.e., *D* = 2. When optimizing three parameters (*D* = 3), we use instead *n* = 20 and *k* = 8 to guarantee a faster exploration of the phase space. For the first generation, we initialize $\vec{q}$ and $\vec{p}$ as null vectors. Moreover, since we do not assume any a priori correlation between the different tuning parameters, the initial form of the covariance matrix Σ is diagonal. Last, all the free parameters *c_i_* of CMA-ES are set equal to 0.2, as proposed in (*16*).

### Simulation details

At every generation, we perform Monte Carlo simulations for each of the sets of parameters drawn from the multivariate Gaussian distribution. In each simulation, after the system has equilibrated, we save 10 independent configurations, which are then used to evaluate the fitness of the samples.

For the HCSS model, simulations are performed in the isobaric-isothermal ensemble in a 2D box with periodic boundary conditions and with a system size of *N* = 256 particles. In all cases, the system is initialized in a disordered, low-density, configuration. The system is equilibrated for 5 × 10^5^ Monte Carlo sweeps, and after that, a total of 10^5^ sweeps are performed, during which we save a configuration every 10^4^ sweeps.

For the SCS model, simulations are performed in both the canonical and isobaric-isothermal ensembles in a 2D box with periodic boundary conditions and with a system size of *N* = 256 particles. In all cases, the system is initialized in a random configuration. The system is equilibrated for 5 × 10^4^ Monte Carlo sweeps, and after that, a total of 10^5^ sweeps are performed, during which we save a configuration every 10^4^ sweeps.

For the 3D system of spherocylinders, simulations are performed in the canonical ensemble considering a system size of *N* = 432 particles in a 3D rectangular box elongated in the *z* direction. In this case, all simulations are initialized in an SM configuration. The system is equilibrated for 5 × 10^4^ Monte Carlo sweeps, and after that, a total of 10^5^ sweeps are performed, during which we save a configuration every 10^4^ sweeps.

## Supplementary Materials

### This PDF file includes:

Sections 1 to 4 Figs. S1 to S4 Table S1
