# Supplementary material for: Inverse design of soft materials via a deep learning–based evolutionary strategy
Source: Sci Adv. 2022 Jan 19;8(3):eabj6731. doi: 10.1126/sciadv.abj6731 (PMC8769546; doi:10.1126/sciadv.abj6731)
Supplement: Supplementary file 1 — Sections 1 to 4 Figs. S1 to S4 Table S1 [file sciadv.abj6731_sm.pdf]

Science Advances
AAAS

Supplementary Materials for

Inverse design of soft materials via a deep learning–based evolutionary strategy

Gabriele M. Coli*, Emanuele Boattini*, Laura Filion, Marjolein Dijkstra

*Corresponding author. Email: g.m.coli@uu.nl

Published 19 January 2022, *Sci. Adv.* **8**, eabj6731 (2022)
DOI: 10.1126/sciadv.abj6731

This PDF file includes:

Sections 1 to 4
Figs. S1 to S4
Table S1

1 Reverse engineering of the QC12 starting from different initial conditions

In the main text, we have shown a trajectory of the reverse engineering of the QC12 in the HCSS model, starting from a specific state point in the region of stability of the fluid phase. Here, we explore whether the performance of the method is affected by a different choice of the initial conditions. To this end, we perform additional trajectories of the reverse engineering of the QC12, starting with a Gaussian distribution centered at different state points, i.e. in the fluid phase, the SQ phase, the HEX phase at relatively high temperature and low pressure, and the HEX phase at relatively low temperature and high pressure.

Supplementary Figure 1 shows the results of the reverse engineering process obtained from four, distinct, initial state points. In all cases, the mean of the parameters distribution converges within the region of stability of the target QC12, clearly showing that the performance is not affected by the particular choice made for the initial conditions.

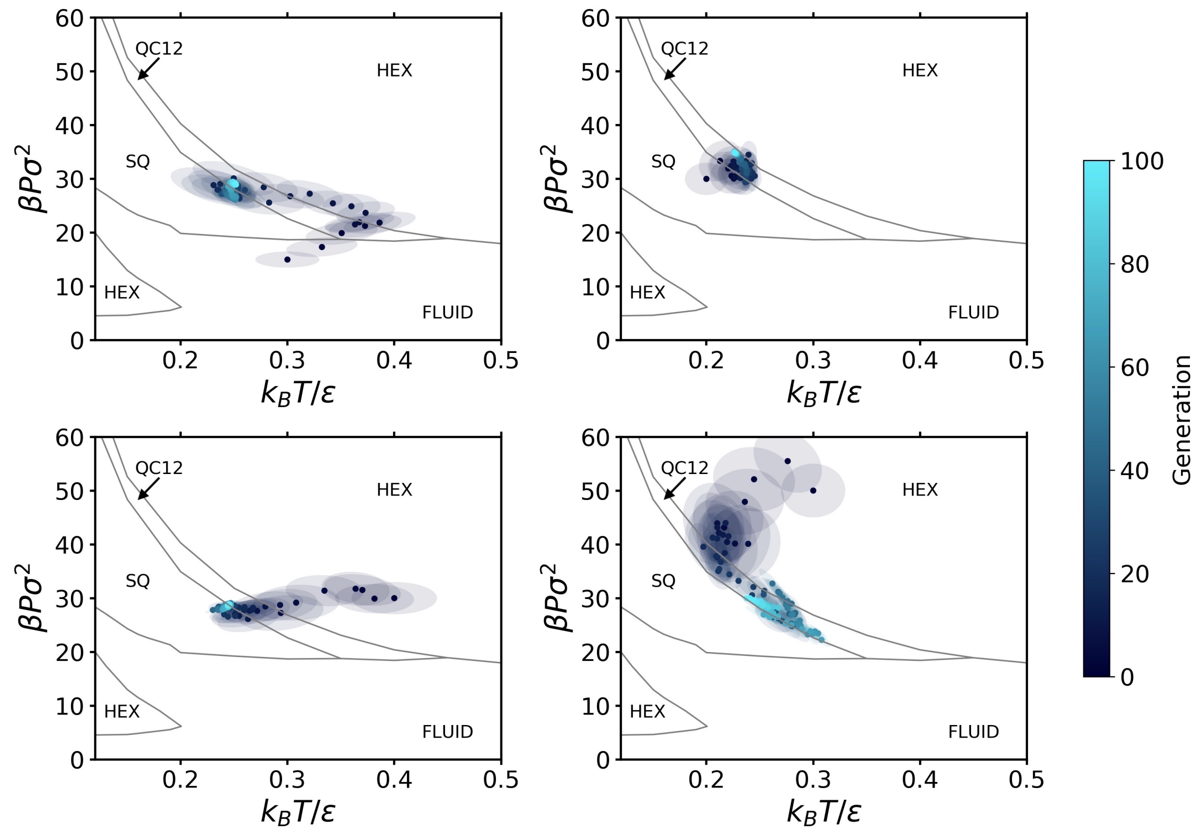

Figure 1: **Reverse engineering of the QC12 starting from different initial conditions**. Four different trajectories showing the evolution of the Gaussian distribution in the $k_BT/\epsilon - \beta P\sigma^2$ plane. Each trajectory, is initialized with the Gaussian centered at different state points, i.e. in the fluid phase (left top), the SQ phase (right top), the HEX phase at relatively high temperature and low pressure (left bottom), and the HEX phase at relatively low temperature and high pressure (right bottom). Points and ellipses represent the mean and the covariance matrix (within one standard deviation) of the distribution. The phase diagram in the background is constructed using data points from Ref. [27].

2 Reverse engineering of the hexagonal crystal in the HCSS model

In the main text, we focused on reverse engineering QCs. However, in principle, the exact same method can be used to reverse engineer any phase that was included in the data set for training the CNN, simply by changing the definition of the fitness. As an example, in Supplementary Figure 2 we report the results of the reverse engineering of the HEX phase in the HCSS model.

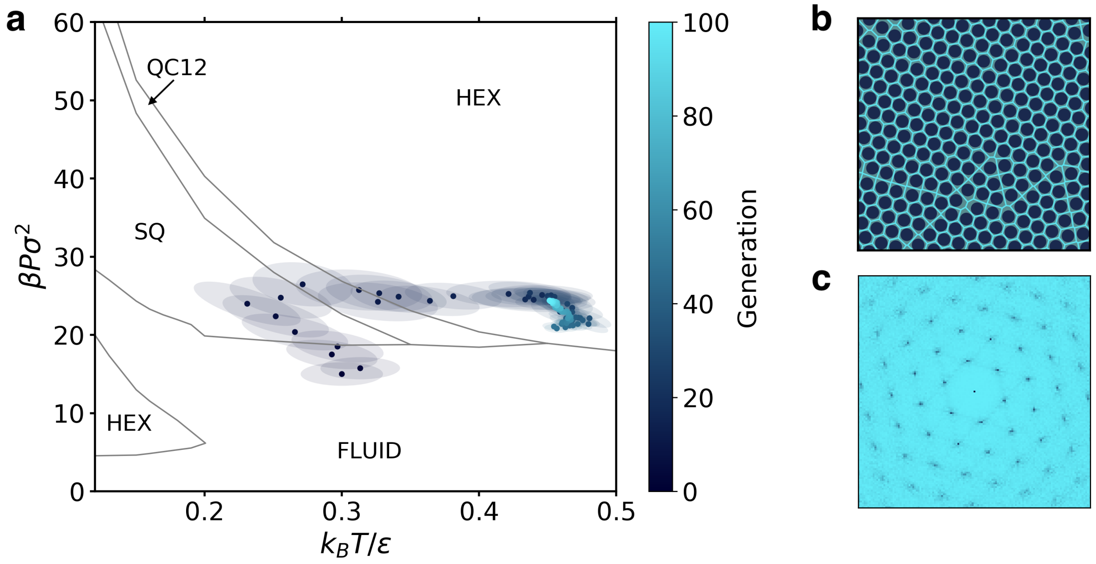

Figure 2: **Reverse engineering of the HEX phase in the HCSS model.** (a) Evolution of the Gaussian distribution in the $k_BT/\epsilon - \beta P\sigma^2$ plane. Points and ellipses represent the mean and the covariance matrix (within one standard deviation) of the distribution. The phase diagram in the background is constructed using data points from Ref. [27]. (b) Representative snapshot of the HEX crystal obtained during the last generation, and (c) its diffraction pattern.

3 Fitness landscape

In order to explain our choice of the fitness function and why it represents an advantageous choice, it is appropriate to separate the discussion into two parts: the first concerns why a

neural network is extremely useful as a fitness function in the context of an IDM compared to, for instance, the BOP-based fitness function used by Coli and Dijkstra in Ref. [12], while the second regards why diffraction patterns were used as input for this neural network.

To answer the first question we consider the case represented in Fig. 4 of the main text, where we reverse-engineered a QC12 phase with the SCS model. In order to show the landscape generated by the fitness function used, we simulate the SCS model on a dense grid of state points, covering the known phase diagram. Calculating the fitness function for each simulated sample (see Fig. 3a), it is evident that the landscape is approximately flat ($f \simeq 0$) in each phase other than QC12. On the contrary, the fitness goes to 1 in the area where the target phase is stable. This is a huge advantage when performing an inverse design process, as it allows the search engine to move freely in the phase diagram. Using a different fitness function, e.g. based on BOPs, this does not happen. In Figs. 3b, 3c, and 3d, we show respectively the two-dimensional BOPs $\bar{\chi}_6$, $\bar{\chi}_7$, and $\bar{\chi}_8$, calculated at the same state points as in 3a. The results show that each order parameter has typical values for each of the phases in the phase diagram. A fitness function which is designed on one or more BOPs typically shows *hills* and *valleys* – i.e. areas of lower and higher fitness value. This can be a relevant problem in order to find the desired phase, which can sometimes be "beyond" a third phase, judged inconvenient to traverse by the search engine. It is in principle possible to combine a high number of BOPs together into a single fitness function, so that the resulting fitness landscape shows only one preferred region, which corresponds to the target phase. This is not a straightforward task though, and it can be easily achieved using a neural network-based fitness function.

Finally, the second question refers to the usefulness of the diffraction pattern as input to the neural network. The reason for this choice is simply due to the fact that this observable contains by definition the totality of the information on the position of the particles in the system. Maintaining a vector nature (and not a scalar one like g(r) or S(k)), it is general enough to describe

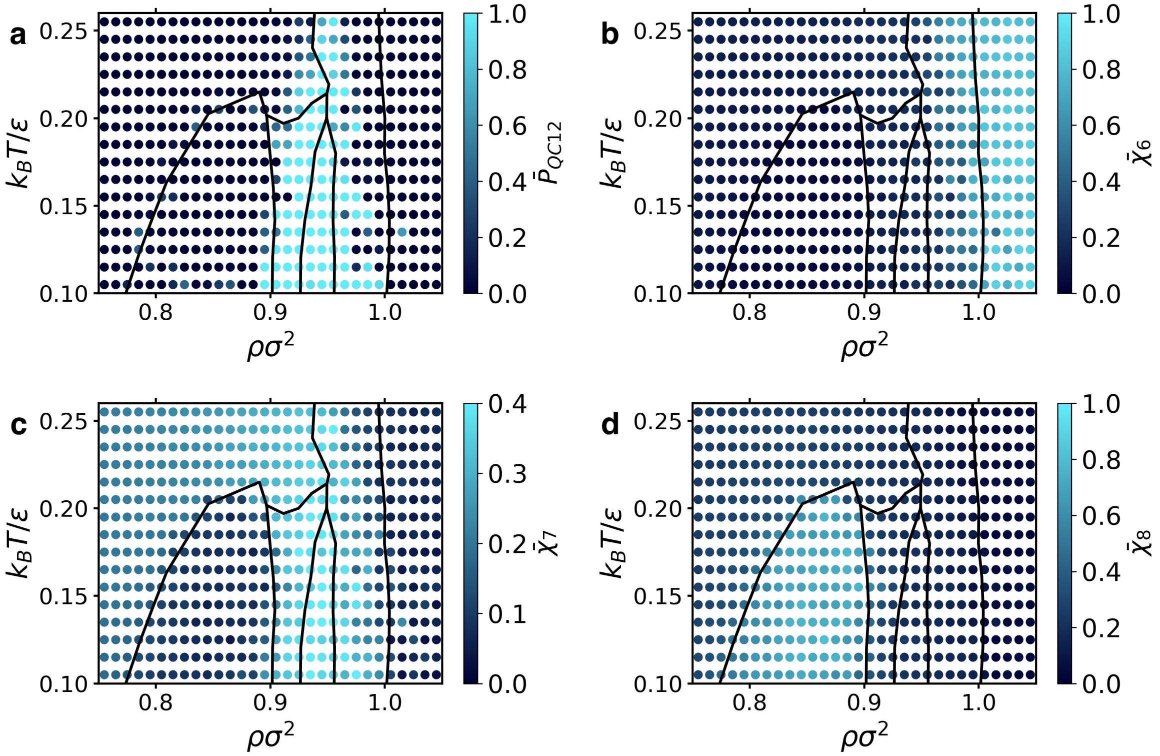

Figure 3: **Order parameters landscapes for the SCS model**. (a) The CNN-based fitness function used in this work, for a dense grid of values on the phase diagram of the SCS model. (b-d) Values of the BOPs $\bar{\chi}_6$, $\bar{\chi}_7$, and $\bar{\chi}_8$, calculated at the same state points as in (a).

even strongly anisotropic phases, such as the system of rods used in this work.

4 Details of the reverse engineering of the QC12 in the SCS model

In the main text, when showing the results of the IDM applied to the SCS model to reverse engineer the QC12, we showed that the algorithm tends to prefer configurations at lower temperature. This raises the legitimate question of whether the CNN gives configurations with less thermal noise a higher score. As we anticipated in the text, this behaviour is a consequence of the CMA-ES equations (see Methods), and in this section we explain how they influence the

$\rho\sigma^2$	k_BT/ϵ	P_{QC12}
0.94	0.12	0.99949
0.94	0.14	0.99961
0.94	0.16	0.99830
0.94	0.18	0.99982

Table 1: Accuracies of the CNN on samples of the SCS model at density $\rho\sigma^2 = 0.94$ for different values of the temperature.

search for the target phase.

In order to shed light on the performance of the CNN in classifying configurations with different degrees of thermal fluctuations, we have simulated the 2D SCS model at several state points, all at density $\rho = 0.94$, and in a temperature range such that each state point is in the region of stability of the QC12. For each of these samples we calculated the fitness function and the results are reported in Tab. 1. What can be seen from the table is that there is no improvement in the fitness function due to a lowering of the temperature. This can also be seen from Fig. 4, in which we plot the maximum fitness recorded at each generation, in contrast to the mean fitness of each generation already shown in Fig. 4. The reason why the mean value of the multivariate Gaussian distribution continues to decrease even after the third generation, where the maximum fitness is already equal to one, is not to be found in a better fitness at low temperatures, but in the updating mechanism of the Gaussian distribution by the CMA-ES algorithm.

When, after the third generation, the mean value of the Gaussian distribution "enters" the QC12 stability region, the covariance matrix of the distribution is still very large, especially along the temperature axis. At each generation, samples are drawn with either lower- or higher-than-average temperatures. The latter, however, will obtain a very low fitness because they are in a region of the phase diagram where the QC12 phase is not stable. Given the CMA-ES equations (see the Methods section), the mean of the Gaussian distribution at the following

generation is given by a weighted sum of the samples we draw, where the weights are given by ranking these samples based on their fitness. This mean is therefore necessarily unbalanced towards low temperatures, and forces the distribution to shift accordingly.

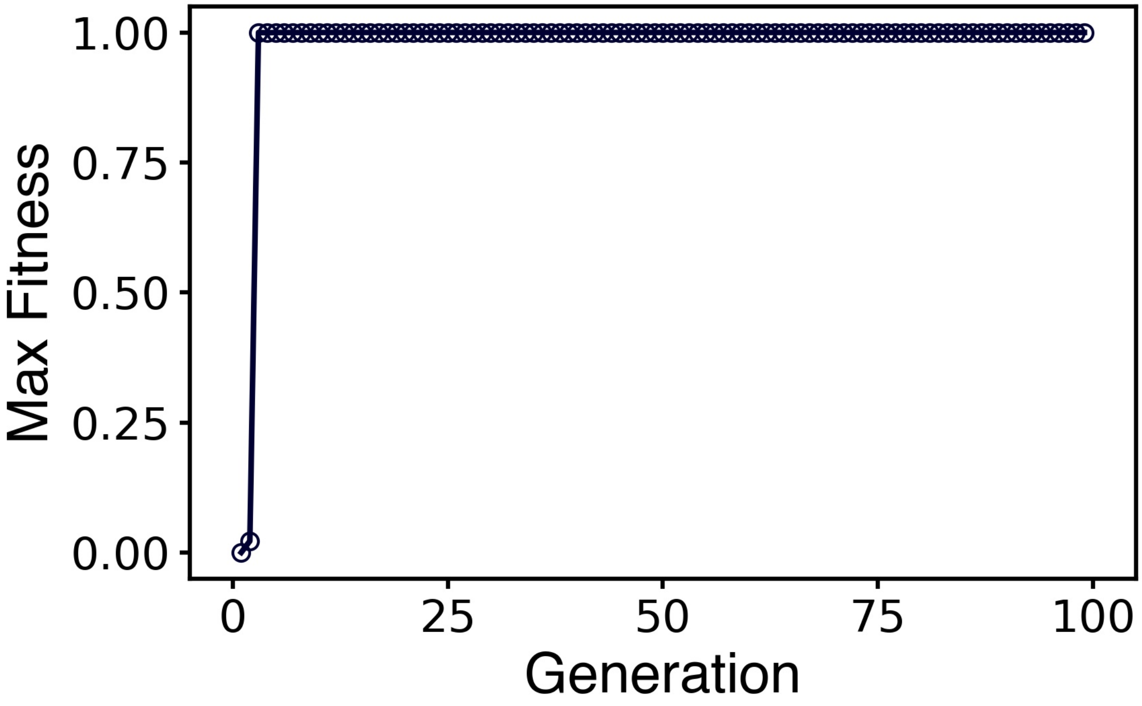

Figure 4: **Evolution of the maximum fitness during the reverse engineering of the QC12 for the SCS model**. Value of the maximum fitness measured at each generation for the trajectory shown in Fig. 4 of the main text. From the third generation, even when the mean value of the fitness is still relatively low (see Fig. 4e of the main text), there is at least one sample whose fitness is very close to one.
